# The Cholinergic System Modulates Memory and Hippocampal Plasticity *via* Its Interactions with Non-Neuronal Cells

**DOI:** 10.3389/fimmu.2017.01489

**Published:** 2017-11-08

**Authors:** Sara V. Maurer, Christina L. Williams

**Affiliations:** ^1^Department of Psychology and Neuroscience, Duke University, Durham, NC, United States

**Keywords:** alpha 7 nicotinic acetylcholine receptor, hippocampal memory, microglia, cholinergic anti-inflammatory pathway, neuroinflammation

## Abstract

Degeneration of central cholinergic neurons impairs memory, and enhancement of cholinergic synapses improves cognitive processes. Cholinergic signaling is also anti-inflammatory, and neuroinflammation is increasingly linked to adverse memory, especially in Alzheimer’s disease. Much of the evidence surrounding cholinergic impacts on the neuroimmune system focuses on the α7 nicotinic acetylcholine (ACh) receptor, as stimulation of this receptor prevents many of the effects of immune activation. Microglia and astrocytes both express this receptor, so it is possible that some cholinergic effects may be *via* these non-neuronal cells. Though the presence of microglia is required for memory, overactivated microglia due to an immune challenge overproduce inflammatory cytokines, which is adverse for memory. Blocking these exaggerated effects, specifically by decreasing the release of tumor necrosis factor α (TNF-α), interleukin 1β (IL-1β), and interleukin 6 (IL-6), has been shown to prevent inflammation-induced memory impairment. While there is considerable evidence that cholinergic signaling improves memory, fewer studies have linked the “cholinergic anti-inflammatory pathway” to memory processes. This review will summarize the current understanding of the cholinergic anti-inflammatory pathway as it relates to memory and will argue that one mechanism by which the cholinergic system modulates hippocampal memory processes is its influence on neuroimmune function *via* the α7 nicotinic ACh receptor.

## Introduction

Cholinergic circuits have been implicated in normal and abnormal cognitive functioning since 1906, when Dr. Aloysius Alzheimer described the symptomatology and neuropathology of the disease that today bears his name (1). Disruption of cholinergic circuitry is likely to be at least partly responsible for the cognitive impairments seen in neurodegenerative disorders (2, 3). Recent studies have also revealed deficits in cholinergic signaling in disorders of attention and cognitive control [see Ballinger et al. (4)]. While the mechanism by which cholinergic signaling influences cognitive processes has been assumed to be direct cholinergic stimulation of pre- and postsynaptic neuronal receptors, a neglected area of investigation is the role of acetylcholine’s (ACh) peripheral and central anti-inflammatory effects on cognition. Neuroinflammation is also a hallmark of neurodegenerative disorders and has been implicated in the neuropathogenesis of Alzheimer’s disease (AD). Not only do neurons respond directly to ACh but also do non-neuronal cells: peripheral macrophages, as well as microglia and astrocytes in the central nervous system (CNS). These non-neuronal cells influence short-term and long-term synaptic function and plasticity [reviewed in Achour and Pascual (5)], and through these mechanisms may contribute to both dysfunction and improvements in cognition.

Though certainly not the only mechanism, this review posits that ACh may influence hippocampal function *via* peripheral and central immune cells, and through this intermediary process, may alter neuronal processes underlying cognition (6). First, this review will describe the intercellular components and pathways of the cholinergic system relevant to memory, with a special focus on the effects of ACh in the hippocampus. Second, this review will explore the role of ACh in hippocampal memory and plasticity, examining both the direct and indirect roles that ACh may have in modulating hippocampal function. A final section of the review will highlight how cholinergic modulation of the immune system may provide new perspectives on regulating memory dysfunction in disease.

## The Cholinergic System

### Synthesis and Synapses

Acetylcholine was first identified by Dale (7) for its actions on heart tissue. It was later recognized as a neurotransmitter by Loewi (8), who initially named it “Vagusstoff” because it was released from the vagus nerve. Since then, the intricate workings of ACh synthesis and synaptic communication have been identified.

Cholinergic synthesis and reuptake in neurons is well understood. First, ACh is synthesized from choline and acetyl-CoA *via* the choline acetyltransferase (ChAT) enzyme. ACh is subsequently transported into vesicles and released into the synaptic cleft, where it can bind to the muscarinic and/or nicotinic ACh receptors. Within the synapse, ACh is broken back down into choline and acetic acid by acetylcholinesterase (AChE). Choline reuptake occurs *via* a high affinity choline transporter, and then choline is recycled in the synthesis of new ACh.

However, neurons are not the only cells to synthesize ACh: cells from the skin, kidney, eye, liver, and placenta all contain ChAT (9). T-cells also show ChAT activity, synthesize ACh, and have been shown to “relay the neural signal” in the cholinergic anti-inflammatory pathway by releasing ACh, which subsequently acts on macrophages *via* the α7 nicotinic ACh receptor (10). ChAT activity has also been found in non-neuronal cells in the CNS, specifically in astrocytes (11). At this time, it is not clear whether microglia show any ChAT activity (12). Further work is needed to pinpoint the cell types involved in ACh synthesis and how they act upon and with neuronal ACh.

### Receptors

There are two kinds of ACh receptors: nicotinic (nAChR) and muscarinic (mAChR). nAChRs, which will be a focus of this review, are ligand-gated ion channels and occur in the neuromuscular junction, autonomic ganglia, and throughout the CNS. One specific subtype of nAChR identified to be functionally important in hippocampal memory [though not the only one; see Chan et al. (13)] is the α7 nAChR.

Using various agonists and antagonists such as nicotine and α-bungarotoxin, nAChRs have been extensively mapped in the rodent brain (14), and, to a lesser extent, the human brain (15). Notably, the hippocampus has almost every nAChR subtype (16), has a high density of α7 nAChR receptors, and expresses cholinergic receptors both pre- and post-synaptically (17). The distribution of receptors is highly preserved across species and is similar in both rodent and human brains (15).

As reviewed in Albuquerque et al. (6), nAChRs are also present in non-neuronal cells, including keratinocytes, endothelial cells, cells in the digestive, respiratory, and peripheral immune systems, and—critically—on glia (18–20). In the brain, both microglia (21, 22) and astrocytes (23) express α7 nAChRs (22, 24).

Alpha-bungarotoxin, which is a specific antagonist to α7 and α9 nAChRs, was used to show the dense nAChR population on human macrophage surfaces (25). Additionally, administration of nicotine decreased α-bungarotoxin binding, further providing support for the specificity of this marker. RT-PCR, western blotting, α-bungarotoxin-conjugated beads, and cloning of cDNA showed definitively that the α7 nAChR was specifically responsible for this binding (25).

### Circuits

In the CNS, cholinergic neurons reside in three major areas: (1) there are cholinergic neurons in the brainstem, where they may function in risk aversion (26). The cholinergic neurons in this area project to and inhibit the thalamus (27, 28). (2) There are cholinergic interneurons in the striatum, which suppress dopamine release (29). (3) There are cholinergic neurons that originate in the basal forebrain, mainly in the medial septum, vertical limb of the diagonal band (MS/VDB), horizontal limbs of the diagonal band, and nucleus basalis. These cells project to the olfactory bulb, neocortex, hippocampus, and amygdala (30–32). Cholinergic neurons in the MS/VDB project to all of the subregions of the hippocampus (33, 34). There are also cholinergic interneurons in the cortex itself, but they are scarce (35). Basal forebrain neurons, specifically those in the nucleus basalis, selectively degenerate in AD (36) and have been a focus of research on the relation between ACh and memory.

There are also projections from the basal forebrain to the frontal cortex, which are involved in attentional processes (37). Attention is known to have a beneficial role in memory itself. Though the interaction of attention and memory is beyond the scope of this review, it is important to note that both functions rely on cholinergic projections from the basal forebrain, so experiments manipulating these connections could be impacting both memory and attention.

Interestingly, few of the recent reviews (38–42) of ACh actions in the CNS mention that many non-neuronal cells in the body and brain manufacture and respond to ACh. As well, few acknowledge that peripheral ACh actions may impact CNS function. These findings are reviewed below.

### ACh in the Periphery

Acetylcholine is the neurotransmitter in all preganglionic neurons in both the sympathetic and parasympathetic nervous systems, as well as all parasympathetic postganglionic neurons. However, only a small number of sympathetic postganglionic neurons are cholinergic (those innervating sweat glands) whereas the rest are adrenergic. Within the somatic nervous system, all motor neurons that innervate skeletal muscles are cholinergic.

#### The Cholinergic Anti-inflammatory Pathway

**Figure 1:**
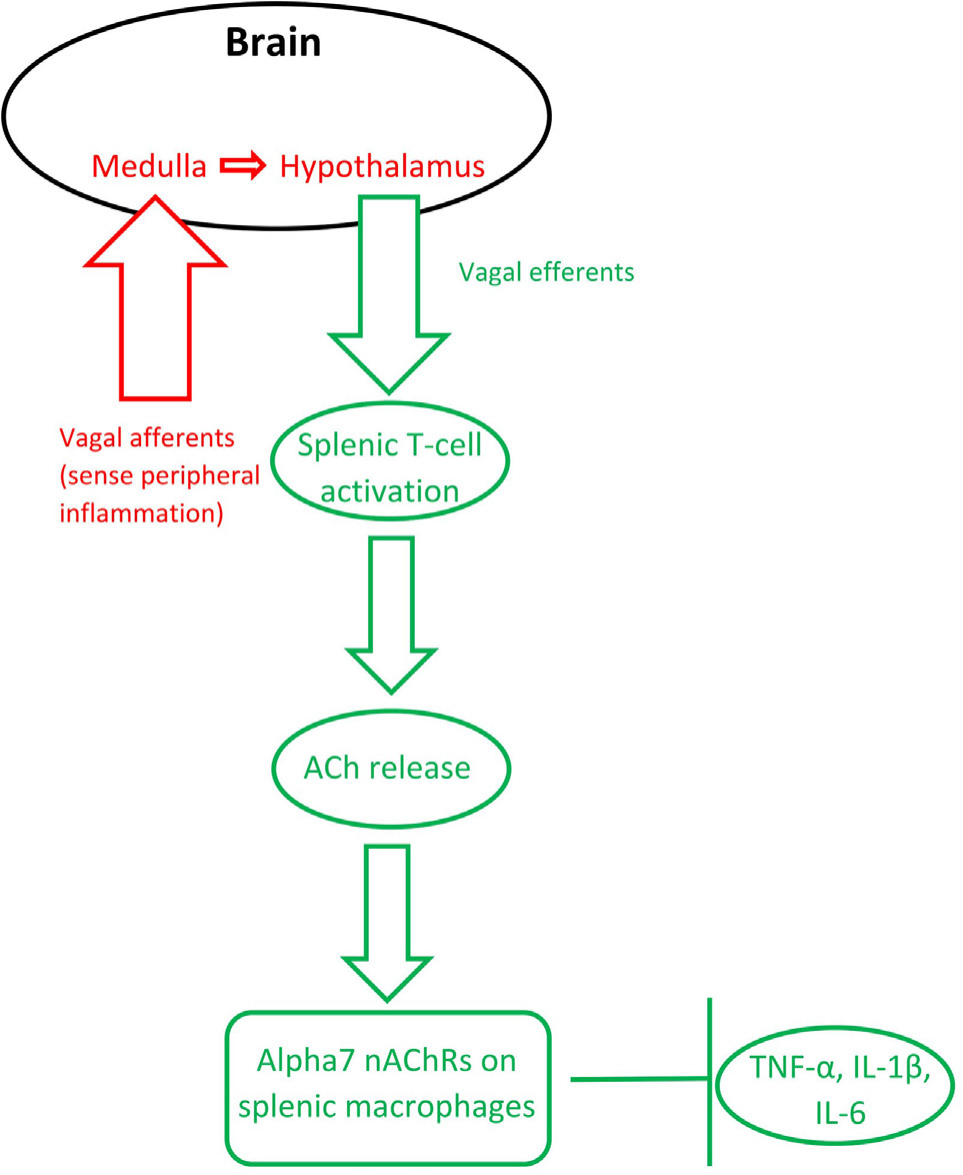
The inflammatory reflex (red) and the cholinergic anti-inflammatory pathway (green). Based on Steinberg et al. (50) and Gallowitsch-Puerta and Pavlov (44).

The finding that activation of the efferent vagus nerve inhibits proinflammatory cytokine release and protects against peripheral inflammation led to this connection between ACh and inflammation to be named “the cholinergic anti-inflammatory pathway” [(43); see Figure 1]. Stimulation of the vagus nerve, either endogenously or through electrical stimulation, leads to increased ACh release (synthesized from T-cells), which acts on macrophage α7 nAChRs. This activation leads to a decreased production of inflammatory cytokines, such as tumor necrosis factor α (TNF-α), interleukin 1β (IL-1β), and interleukin 6 (IL-6), by macrophages [reviewed in Gallowitsch-Puerta and Pavlov (44)]. Peripheral ACh also acts on α7 nAChRs on lymphocytes to suppress inflammation (20, 45–47). ACh also produces a dose-dependent inhibition of IL-6, IL-1β, and TNF-α production in human macrophages [reviewed in Pavlov and Tracey (43) and Borovikova et al. (48)] and in whole-blood of rheumatoid arthritis patients (49), whereas vagotomy leads to increases in IL-6 and, to a lesser extent, TNF-α (48).

Importantly, the vagus nerve’s anti-inflammatory action is also bidirectional: the afferent vagus nerve detects peripheral cytokines, then communicates through the medulla to the hypothalamus, which subsequently communicates *via* the efferent vagus nerve to inhibit inflammation in the periphery (51). In other words, the bidirectional anti-inflammatory communication between the brain and periphery relies on the vagus nerve and ACh signaling. Termed the “inflammatory reflex,” the afferent vagus immediately regulates production of pro-inflammatory cytokines to avoid overproduction (52).

The cholinergic anti-inflammatory pathway relies on activation of α7 nAChRs on macrophages. Alpha7 nAChR stimulation has been shown to modulate TNF-α release (53). AChE inhibitor administration, which decreases the breakdown of ACh, leads to lower levels of IL-6 and TNF-α, but does not significantly alter cytokine release in α7 nAChR knockout mice (54). Macrophages stimulated with the bacterial mimic lipopolysaccharide (LPS) produce more TNF-α, which is blunted by nicotine pretreatment; however, additional pretreatment with an antisense oligonucleotide for α7 nAChRs (but not α1 or α10 nAChRs) ameliorated this effect (25). These findings specifically implicate the α7 nAChR, a receptor otherwise known to be important for hippocampal memory, in the cholinergic anti-inflammatory pathway.

Like peripheral macrophages, microglia have α7 nAChRs, which, when activated, suppress pro-inflammatory cytokine release (53, 55). In mouse and human cell culture studies, it has been shown that α7 nAChRs on microglia are necessary for blunting TNF-α and downstream IL-1β production (55, 56). AChE inhibitors have been shown to suppress TNF-α secretion from microglia, and addition of α-bungarotoxin blunted these effects (57). Peripheral macrophages and microglia—the macrophages of the brain—seem to respond similarly to ACh.

Thus far, this review has summarized evidence that ACh is synthesized by neurons but also by non-neuronal cells. Basal forebrain cholinergic neurons release ACh in all regions of the hippocampus, which all contain nAChRs. The hippocampus in particular is a region with a high density of microglia and astrocytes (58) as well as a high density of nAChRs. These findings suggest that cholinergic stimulation of the hippocampus not only has direct neuronal effects but also effects on microglia and astrocytes that may modulate neuronal function.

## Memory and Neural Plasticity

### The Classic View of the Cholinergic System in Memory

Recent reviews of basal forebrain cholinergic systems in memory and cognition (4, 59–62) focus on the septohippocampal pathway, which is widely known to be implicated in memory processes. Below, the standard view of septohippocampal ACh functions in memory are reviewed, followed by a proposal for some alternate means by which ACh may have effects on hippocampal memory.

There are a number of lines of evidence that support the view that hippocampal ACh is important for memory [see Parent and Baxter (63)]. First, during spatial memory tasks, cholinergic markers such as ChAT are upregulated [see Park et al. (64)]. Second, ACh levels in the hippocampus are correlated with memory function. For example, there is a correlation between age-related cognitive decline and decreases in hippocampal ACh (65). Multiple studies find a correlation between spatial memory and ACh release both in the hippocampus (66), and within the basal forebrain (67). Also, damage to the septum leads to decreases in both spatial memory performance and hippocampal levels of ACh (68). Function can be rescued when basal forebrain AChE is inhibited pharmacologically. Third, both the direct infusion of ACh into the hippocampus and direct pharmacological activation of nAChRs in the hippocampus reverse the cognitive deficits caused by damage to the septum (69–72). Importantly, this finding shows that while the basal forebrain provides multiple inputs to the hippocampus, direct activation of nAChRs in the hippocampus reverses cognitive dysfunction caused by interruption of this pathway. While all of these data have traditionally been interpreted as direct actions of ACh on neuronal receptors, hippocampal astrocytes (18) and microglia (53) also express nAChRs. Therefore, all of these findings leave open the possibility that some of the actions of ACh on the hippocampus may be *via* nicotinic activation of glial cells. However, more research to tease apart the direct neuronal and indirect non-neuronal actions of ACh in memory is needed.

#### ACh is the “Decider” between Encoding and Retrieval

The classically held view is that ACh is the decider between encoding mode and retrieval mode in memory processing (73, 74). ACh is associated with suppressing old associations and inhibiting proactive interference. Rats with cholinergic basal forebrain lesions perform comparably to controls in a water maze task unless the location of the platform changed daily (75). An explanation for this finding is that the lack of ACh in the hippocampus leads to more expression of a previously encoded association (which would be the previous location of the platform). However, rats with intact cholinergic systems are able to inhibit the previous association, and form a new one. Hasselmo (74) in addition to Easton et al. (73) provided extensive reviews describing what may be the neural underpinnings of this phenomenon. Briefly, the CA1 region of the hippocampus receives input from two brain regions: entorhinal cortex layer 3 (associated with sensory perception—“extrinsic input”) and the CA3 region of the hippocampus (associated with previously formed associations—“intrinsic input”). ACh reduces the relative input from CA3, hence allowing sensory inputs to be encoded, free from proactive inhibition. In this way, hippocampal ACh “prioritizes encoding” in novel contexts. This extended model implicates hippocampal ACh directly in the encoding phase and also allows working memory to be more efficient.

Further support for this hypothesis mostly relies on hippocampal ACh and its association with increased exploratory behavior. Evidence shows that hippocampal ACh levels are increased in instances of novelty (76) and exploratory rearing (77) compared to non-novel environments. The authors conclude from this work that the state of novelty itself activates the cholinergic system. Frontal cortical and dorsal hippocampal ACh, glutamate, and GABA measured through microdialysis in response to exploration of a novel environment support this interpretation (76). In both brain areas, but exaggerated in the hippocampus, ACh was significantly increased during the first session of exploration. Additionally, infusion of ACh agonists into the hippocampus led to increased exploratory behavior (78). The take-away from these studies is that increased hippocampal ACh release, caused by a novel environment, aids encoding and increases exploratory behavior. While this evidence points to hippocampal ACh as an indicator and facilitator of encoding in novel contexts, it does not address the contribution of other cells that ACh may stimulate.

### Non-Neuronal Actions of the Cholinergic System in Memory

**Figure 2:**
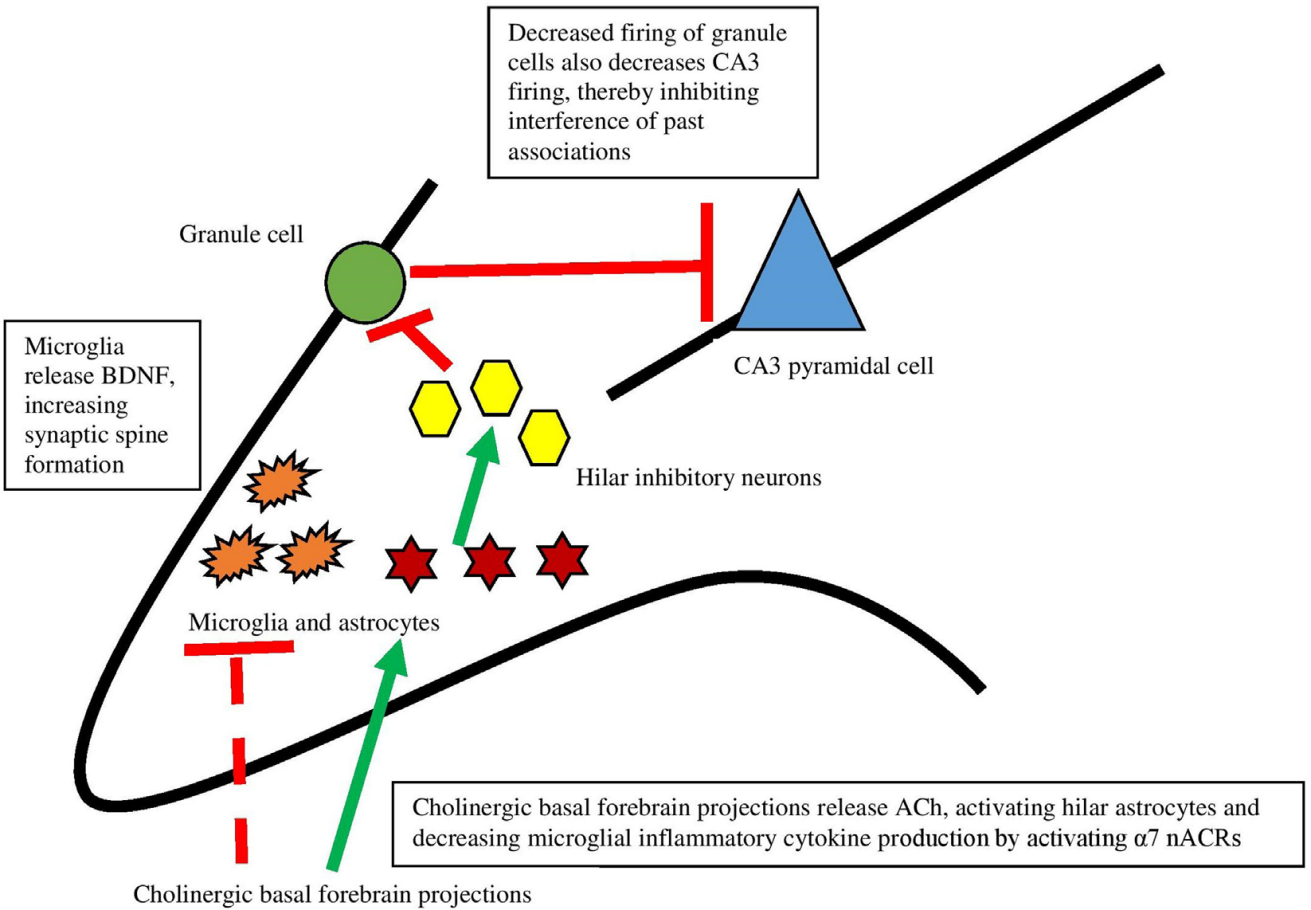
The interaction between the cholinergic system and glia and its impact on the hippocampus. Dotted lines represent cytokine effects, and solid lines represent cell activation/firing. Cholinergic basal forebrain projections release ACh and both decrease cytokine release from microglia and activate hilar astrocytes. Those astrocytes activate inhibitory interneurons, which decreases firing from granule cells. This decreased firing leads to decreased firing of CA3 pyramidal cells, preventing interference of past associations in encoding. Based largely on Hasselmo (74) and Pabst et al. (79).

Recent work has, in fact, demonstrated that ACh acts directly on hippocampal astrocytes, which then leads to alterations in firing of hippocampal neurons (79). Consistent with Hasselmo’s view (80) that high levels of ACh aid encoding by suppressing inappropriate activations, specific optogenetic stimulation of septal cholinergic neurons led to decreased firing of dentate granule cells. Administration of an α7 nAChR antagonist blocked this effect, indicating that this receptor is responsible for the decreased firing. The critical aspect of the findings, however, is that disrupting astrocytic function in the hilar layer of the hippocampus by an inhibitor of glial metabolism prevented inhibition of dentate granule cells caused by septal cholinergic stimulation. These findings revealed that septohippocampal release of ACh causes a slow inhibition of dentate granule cells, not by acting directly on neurons but rather by activating astrocytes (see Figure 2). As well, it was found that a nAChR antagonist prevented activation of astrocytes, indicating that astrocytes are specifically responding to basal forebrain cholinergic inputs through this class of receptor, specifically the α7 nAChR. When activated, astrocytes can release neurotransmitters like glutamate (81, 82) and consequently suppress activation of dentate granule cells *via* hilar inhibitory interneurons. As further support for the view that hippocampal astrocytes play a role in hippocampal function, blocking transmitter release from astrocytes impaired hippocampal-dependent novel object recognition memory, but not all types of memory (83). These data provide support for the view that basal forebrain ACh release may influence hippocampus and memory *via* slow inhibition of neuronal activity *via* astrocyte intermediaries.

In addition to astrocytes, microglia are necessary for hippocampal memory and motor-learning dependent synapse formation, likely *via* microglial brain-derived neurotrophic factor (BDNF) (84). To address whether or not microglia are necessary for memory, mice expressing tamoxifen-inducible Cre recombinase that allowed for specific manipulation of gene function in microglia were generated. Then, Cre was used to induce diphtheria toxin receptor expression solely in microglia. Mice depleted of microglia *via* diphtheria toxin administration showed deficits in hippocampal-dependent fear conditioning and novel object-memory tasks, indicating that microglia are critical for emotional and hippocampal-dependent memory (85). As well, depleting microglial BDNF mimicked the effects of microglial elimination on memory function and synaptic spine formation, suggesting a potential mechanism for the memory effects. Interestingly, there were no differences in the expression of TNF-α, IL-1β, and IL-6 in microglial-depleted and control mice—possibly indicating a compensatory production mechanism (for example, production of cytokines by non-microglial cells like astrocytes, or cytokines entering the CNS through the blood–brain barrier). Regardless, the memory impairments were not due to the increase or decrease of these cytokines. Microglial depletion has also been shown to impair spatial memory in mice trained on a Barnes maze (86).

Though microglia are necessary for memory, overactivation of microglia, which causes increased release of proinflammatory cytokines (such as TNF-α, IL-1β, and IL-6), is detrimental. This has been demonstrated in several studies of postoperative cognitive dysfunction (POCD).

Postoperative cognitive dysfunction is a decline in memory and executive functions that occurs shortly following surgery in some patients and can persist for several months or more (87). Memory impairment also occurs following peripheral orthopedic surgery in mice (88, 89). This mouse model of POCD has revealed that surgery not only increases pro-inflammatory cytokines in the periphery but also upregulates inflammation in the brain (90). These effects are not due to anesthesia, as mice given anesthesia without surgery do not show this inflammatory profile (90). Macrophage-produced TNF-α appears to promote POCD by altering the permeability of the blood-brain barrier, allowing increased macrophage infiltration to the hippocampus after orthopedic surgery (88): prevention of macrophage-produced TNF-α prevented the increased permeability of the blood–brain barrier, and subsequently also prevented the increased macrophage migration into the brain after surgery. Orthopedic surgery leads to increased IL-1β in the hippocampus and impaired hippocampal-dependent fear conditioning, while anti-TNF antibody administration inhibits this effect (90).

Interestingly, stimulation of α7 nAChRs prevents the macrophage migration and cognitive deficits seen after surgery, and administration of an α7 antagonist increased neuroinflammation and POCD (88, 89). Because one of the agonists, choline, does not easily cross the blood–brain barrier, the authors conclude that it must be acting on peripheral macrophages. The cholinergic anti-inflammatory pathway takes effect both peripherally by acting on macrophages to decrease proinflammatory cytokine release, and centrally by decreasing hippocampal pro-inflammatory cytokines (89) and macrophage activation and migration. Both of these inflammatory metrics influence fear conditioning: surgery leads to impaired memory, but α7 agonists rescue this behavior. Alpha7 antagonists further impair memory after surgery. Notably, orthopedic surgery also decreases hippocampal BDNF and neurogenesis (91).

In a study examining the effect of minocycline administration and surgery on inflammation and memory in an aged-mouse model of POCD, minocycline (which decreases microglial activation, but may also have effects on neurons) administered prior to surgery not only decreases hippocampal levels of inflammatory cytokines TNF-α, IL-1β, and interferon-γ but also rescues spatial memory deficits that occur following surgery (92). Previous findings showing that microglia have α7 nAChRs suggest that activation of the cholinergic anti-inflammatory pathway might have similar effects to treatment with minocycline. The authors suggest that microglia may “get stuck” in an activated state after an immune challenge, and the continued release of inflammatory cytokines might facilitate cognitive aging and the associated memory impairment.

There is considerable support for the important role of cytokines released from microglia in memory modulation. Administration of inflammatory cytokines (specifically, TNF-α and IL-1β) causes deficits in spatial memory (93–95) and hippocampal-dependent fear conditioning [(96); for review, see Pugh et al. (97)]. Following infection, it appears that it is microglia, not neurons or astrocytes, in the hippocampus that are responsible for the increase in IL-1β, which then leads to impaired memory (98). Blocking IL-1β release in the CNS prevents the memory impairment caused by overactivation of microglia (99). Importantly, the complete absence of IL-1β is detrimental to memory—indicating basal levels are necessary, but overexpression of the cytokine is harmful (98). Even in the absence of other inflammatory factors and insults, the increased expression of TNF-α led to decreased performance in memory tasks such as passive avoidance (100). Though there is much research on the impact of inflammation in memory, more work is needed on the integration of ACh and neuroimmune factors on hippocampal-dependent memory.

### Markers of Plasticity: Neuronal and Non-Neuronal Influences

As described previously, basal forebrain cholinergic inputs to the hippocampus play an important role in cognitive function. Therefore, it is not surprising that there is considerable evidence that ACh receptors are involved in various aspects of neural plasticity: long-term potentiation (LTP), regulation of BDNF, and hippocampal neurogenesis.

#### Long-term Potentiation

Acetylcholine “biases the system” toward increased LTP, believed to be one of the cellular foundations of learning and memory, by decreasing the induction threshold required (101, 102). In addition, in an *in vitro* high ACh environment, stimulation that normally produces long-term depression produces LTP (103). The specific neuronal mechanisms underlying this effect have largely been identified. ACh, when it binds to a muscarinic ACh receptor, leads to a signaling cascade activating phospholipase-C, which has been shown to contribute to LTP (104). Additionally, impaired LTP has been linked to malfunctioning α7 nAChRs (105). A blockade of α7 nAChRs blunted LTP, and α7 nAChR knockout mice can similarly show decreased LTP (106). The cholinergic systems’ impact on LTP has always been interpreted as a direct synaptic action, but it is also possible that ACh is acting on glial α7 nAChRs, though research to date has not demonstrated this conclusively.

Impairments in hippocampal LTP have been linked to microglial overactivation, and minocycline normalizes these impairments (107). Clearly, microglia have an important role in LTP that has not been explored fully, and this role is possibly mediated by microglial α7 nAChRs.

Pharmacological activation of α7 nAChRs leads to increases in hippocampal LTP, quantified by long-lasting increases in calcium activity in the CA1 and CA3 regions of the hippocampus in wild-type, but not α7 nAChR knockout mice (108). Because the α7 nAChR is highly permeable to calcium, this specific receptor is likely causal for this effect (109). These data support the view that α7 nAChR activation on neurons as well as on microglia both aid LTP.

The effect of α7 nAChR activation is usually the decreased release of inflammatory cytokines, and these molecules have also been shown to impact LTP. TNF-α directly modulates the strength of synapses by altering postsynaptic AMPA receptor expression (110), leading to weakened synaptic strength and increased likelihood for that synapse to be engulfed by activated microglia. A proposed mechanism that may explain cognitive dysfunction in patients with immune disorders is suppression of the cholinergic anti-inflammatory pathway leading to heightened secretion of TNF-α, altering astrocyte-neuron signaling, leading to a cascade resulting in a restructuring of the excitability of hippocampal synapses (111).

During LTP and hippocampal memory tasks, hippocampal IL-1β is released, and blocking IL-1 receptors has an adverse effect on both memory and LTP (98, 112). So, though IL-1β is required for LTP, overexpression—such as that seen in pathological conditions—inhibits LTP (113). In other words, the effects of IL-1β on LTP follow the same “U-shaped” concentration-response curve as the effects of IL-1β in memory: moderate levels are necessary, but overexpression is detrimental. Because ACh inhibits release of inflammatory cytokines, perhaps the impact of ACh on LTP occurs *via* both direct neuronal and indirect non-neuronal action.

#### Brain-Derived Neurotrophic Factor

Acetylcholine has been shown to modulate plasticity *via* BDNF. Following chronic nicotine exposure (which activates nAChRs), BDNF in the hippocampus is upregulated (114, 115). Conversely, after loss of basal forebrain cholinergic neurons, hippocampal BDNF subsequently decreases (116). These data have been interpreted as a direct effect of ACh on neurons and this view is supported by the finding that in cultures of cortical neurons, α7 nAChR stimulation produces dose-dependent increases in BDNF. However, there is also evidence that microglia modulate BDNF release. For example, hippocampal microglial activation following LPS administration led to decreased BDNF in the CA1 region of the hippocampus (117). Two studies by Ruth Barrientos and colleagues show that hippocampal IL-1β, likely produced by microglia, regulates BDNF. In one study, social isolation stress lowered BDNF levels in the hippocampus of mice, but levels were restored when an IL-1 receptor antagonist was administered to the hippocampus (118). In a second study, the increase of hippocampal BDNF following context learning was blocked by administering IL-1β to the hippocampus (119). High levels of IL-6 also suppress BDNF (120). There is at least some evidence that ACh modulation of inflammation can alter BDNF release. Vagal nerve stimulation, known to release ACh in the periphery and to be a catalyst for the cholinergic anti-inflammatory pathway, has been shown to upregulate both neurogenesis and BDNF in the hippocampus after 24 hours and 3 weeks of treatment (121). Though the mechanism of this effect is not yet known, it may be because vagal stimulation also enhances serotonin (*via* the raphe nucleus) and norepinephrine (*via* the locus ceruleus) in the CNS [see Biggio et al. (121)]. This increase in norepinephrine could lead to increased cholinergic signaling in the basal forebrain (122), contributing to increased hippocampal neurogenesis (123). Peripheral cholinergic stimulation (89) and vagal nerve stimulation (124) are also known to decrease neuroinflammation, which contributes to increased hippocampal neurogenesis (125).

Importantly, the effects of BDNF and activation of α7 nAChRs appear to be reciprocal. BDNF increases the density of α7 nAChRs on hippocampal neurons (126), and activation of α7 nAChRs leads to upregulated BDNF in the hippocampus (127). Together, these data suggest that the cholinergic system modulates BDNF and neural plasticity *via* both direct neuronal and indirect glial actions.

#### Neurogenesis

The dentate gyrus (DG) of the hippocampus is one of the most plastic regions in the mammalian brain because it is able to generate principal neurons that integrate into the pre-existing network throughout life. Moreover, basal forebrain cholinergic projections to the DG have been shown to facilitate neurogenesis (128–130). Enhanced adult hippocampal neurogenesis improves pattern separation ability, temporal separation of events in memory, forgetting, and cognitive flexibility [see recent review: Hvoslef-Eide and Oomen (131)]. Because these abilities rely on suppressing older memories and inputting new associations, they likely rely on cholinergic inputs that modify hippocampal neurogenesis.

Neural stem cells in the hippocampus express ACh receptors, including mAChRs and α7 nAChRs (130), providing a possible mechanism by which the cholinergic system influences neurogenesis. In general, *in vivo* and *in vitro* studies show that cholinergic receptor stimulation increases neural stem cell proliferation (132). Activation of the α7 nAChR *via* increased ACh levels has been shown to enhance new neuron survival, but not differentiation or proliferation [see Kita et al. (133) and Narla et al. (134) for reviews]. These manipulations may be influencing neuronal progenitors through ACh receptors, but they also may be impacting neuronal proliferation and survival by acting on microglia. However, this possibility has not been addressed directly.

Cholinergic stimulation *via* increased ACh levels also promotes hippocampal neurogenesis, and decreased ACh levels impair it (123, 135). There is some evidence that a high-choline diet in adulthood, leading to increased ACh synthesis (136), increases proliferation and/or survival of hippocampal neurons (137). If choline-induced proliferation occurred, then it was likely due to a different mechanism than the α7 nAChR because of previous research implicating this receptor in neuronal survival but not proliferation [see Kita et al. (133) and Narla et al. (134) for reviews]. AChE inhibitors also upregulate proliferation of cells in the DG (129, 130, 132) and exercise-induced proliferation of aged neural stem cells is prevented by lesions of the septal cholinergic system (132, 138). These findings indicate that neural stem cells respond to cholinergic stimulation even in aged animals.

As was true for memory function, the immune system is needed for neurogenesis, but overactivation leads to a decrease in neurogenesis. Mice lacking T- and B-cells have impaired hippocampal neurogenesis, which is rescued by reintroducing T-cells in the periphery (139). Because some of the reintroduced T-cells likely had the ability to make and release ACh, it is possible that the restoration of neurogenesis was *via* increases in ACh. As with LTP, DG neurogenesis requires some microglia (140). However, microglial overactivation, for example following stress, infection, or disease, appears to compromise neurogenesis, which may contribute to the memory impairments seen in these conditions. When the immune system is activated by stress, minocycline decreases microglial activation and rescues adult hippocampal neurogenesis in mice (141). Minocycline also rescues neurogenesis in AD-model and schizophrenia-model mice that have been exposed to LPS (142–144). IL-6 and TNF-α have been shown to decrease hippocampal neurogenesis in adulthood when overexpressed (125), providing further evidence that an increase in neuroinflammation leads to a decrease in neurogenesis.

In addition to impairing neurogenesis, inflammatory cytokine IL-6 is neurotoxic. In a study analyzing amyotrophic lateral sclerosis (ALS) blood cells, which normally secrete TNF-α and IL-6, researchers found that these cells were toxic to rat neurons *in vitro* (145). Adding an anti-IL-6 antibody blunted the toxicity, and anti-TNF-α and anti-IL-1β antibodies did so to a lesser extent. This study in particular is critical in parsing out the effects of the often-grouped-together cytokines (TNF-α, IL-1β, and IL-6): IL-6 is shown to play the largest role of the three in neurotoxicity. Additionally, overexpression of IL-6 led to more neurodegeneration by several metrics (146). Inflammatory cytokines released by microglia lead to impaired neurogenesis and increased neurodegeneration. There have not been specific studies elucidating the role of microglial α7 nAChRs in neurogenesis, but it is possible that their activation leads to fewer inflammatory cytokines, leading to increased neurogenesis and cell survival. Together, these data suggest that cholinergic stimulation of the DG may modify hippocampal neurogenesis directly *via* stimulation of ACh receptors on DG stem cells or indirectly *via* stimulation of microglia and the subsequent inhibition of IL-6 and TNF-α.

## Clinical Applications

Inflammation and the central release of inflammatory cytokines have been proposed as a mechanism underlying cognitive decline or dysfunction in mouse models of AD [reviewed in Mosher and Wyss-Coray (147)], stress (148), cancer-treatment (e.g., irradiation and chemotherapy) (149, 150), multiple sclerosis (151), and obesity (152). The degeneration of the basal forebrain cholinergic system is a factor in many forms of dementia: not only in AD, but also in Parkinson’s disease, Down syndrome, ALS, and supranuclear palsy [see Ferreira-Vieira et al. (60)]. One likely possibility that is sometimes overlooked is that the loss of cholinergic input in these disorders may unmask inflammation in the hippocampus, leading to impaired cognition.

### Parkinson’s Disease

Parkinson’s disease, while characterized by motor deficits, produces impairments in working memory, attention, and a two to six times higher risk of dementia (153, 154). Mouse models show decreased cortical ACh and impaired performance in hippocampal tasks such as the Y-maze, novel-object recognition, object-place recognition, and operant reversal learning (155). Activation of nAChRs, including the α7 nAChR, is neuroprotective in animal models of Parkinson’s disease [reviewed in Quik et al. (156)]. In Parkinson’s patients, lower cholinergic activity, lower basal forebrain volume, and cholinergic denervation in the thalamus and cortex are all associated with impaired cognition (38, 157–159). Based on this evidence, the cholinergic deficits underlying Parkinson’s disease may be causally linked to the memory deficits.

While Parkinson’s disease causes a loss of dopaminergic neurons, it also impacts the cholinergic system and produces neuroinflammation. For example, in a mouse model of Parkinson’s, microglia and astrocytes in the substantia nigra are increased in number and show more activation-associated morphology compared to controls (24). Microglial number, as well as astrocyte number and activation, were decreased following application of a nAChR agonist. Alpha7 nAChR activation prevents dopaminergic cell death by inhibiting the activation of both astrocytes and microglia in the substantia nigra (24). These findings suggest that Parkinson’s is inherently inflammatory, and this inflammation is decreased by activating nAChRs. This inflammatory profile may be either the cause or a result of dopaminergic cell death. If nAChR activation, and hence activation of the cholinergic anti-inflammatory pathway in the brain, prevents dopaminergic cell death, it is possible that the inflammation itself has a role in neurodegeneration in Parkinson’s. Therefore, blunting neuroinflammation by activating α7 nAChRs might be a promising therapy to prevent neuron loss in these patients. Though this study examines the substantia nigra, it is possible that inflammation is also occurring in the hippocampus, which would then contribute to loss of cognitive function seen in Parkinson’s.

Cholinergic drugs have, in fact, been used to treat Parkinson’s-associated cognitive deficits with some success [see Pagano et al. (160) for a meta-analysis]. Interestingly, the prevalence of Parkinson’s disease is decreased in smokers [see Quik et al. (161)] suggesting that nicotine may be neuroprotective, though the target of nicotine action for this beneficial effect is not yet known [see Piao et al. (162)]. It has been suggested that neuroinflammation may not just be the result of the cell loss seen in Parkinson’s disease, but that noradrenergic and cholinergic hypofunction may contribute to dysregulation of neuron-glia interactions, leading to inflammation and, eventually, neurodegeneration (163). Thus, a better understanding of the role of cholinergic systems in modulating communication between glia and neurons may lead to the development of cholinergic drugs that could be promising for prevention or treatment of Parkinson’s disease.

### Alzheimer’s Disease

Cholinergic neurons in the basal forebrain selectively degenerate in AD (36, 164, 165), and these cells are also the first neurons effected in early AD [see the following reviews (40, 166, 167)]. Impaired cognitive function in AD is associated with increased neurofibrillary tangle density in the basal forebrain (168). As well, postmortem brains of AD patients have lower levels of ChAT and AChE, regardless of age (169). These findings led to the “cholinergic hypothesis” for AD and to the treatment of AD patients with AChE inhibitors. The widely held view is that this drug treatment helps to negate the loss of cholinergic neurons by increasing ACh postsynaptic action. In contrast, not much work has been devoted to the effects of these drugs on glia in AD patients (170).

AD is also characterized by a heightened profile of neuroinflammation [see Calsolaro and Edison (171)]. AD patients have more TNF-α and IL-6 in both serum and brain, indicating an inflammatory phenotype (172, 173), and higher levels of TNF-α are highly correlated with rapid cognitive decline (174). Post-mortem AD brains also have more activated microglia and astrocytes (175). Microglia in particular have been implicated in the excessive neuronal loss seen in AD (176) and overactivation of microglia is seen relatively early in the progression of the disease (177). Thus, AD neuropathology is characterized not only by a loss of cholinergic neurons but also by increased neuro-inflammation. Interestingly, peripheral inflammation has been linked with early-onset AD more so than with late-onset type AD (178), suggesting that inflammation may be more critical feature in the development or progression of the familial form of the disease.

As in Parkinson’s disease, it is still not clear exactly how the innate immune system is involved in AD, specifically whether it is a cause or effect of pathology. Some studies show that neuroinflammation, though contributing to an AD phenotype, is initiated by amyloid-β and thus occurs as a result of AD neuropathogenesis. For example, microglial TNF-α was shown to catalyze a cascade of cellular events *in vitro* that characterize AD, including cell toxicity (179). Amyloid-β peptides also appear to activate microglia, which initiate the toxic “cell cycle events” *via* TNF-α. In contrast, TNF-α knockout mice do not show these toxic “cell cycle events”—powerfully implicating this cytokine in the neuronal death seen in AD. Because there are α7 nAChRs on microglia, perhaps activation of these receptors could have a hand in preventing AD pathology.

It is worth mentioning that the interaction between the cholinergic and neuroimmune systems differs in AD versus normal aging [see Schliebs and Arendt (180)]. Specifically, cholinergic cell *loss* is seen in AD, but cholinergic *dysfunction* (in the form of synaptic losses and other modifications) is seen in normal aging (36, 181–183).

However, the link between TNF-α, IL-1β, and IL-6 and AD is not completely clear. Expression of these cytokines in blood serum is increased in early-onset AD patients, but not late-onset AD patients (178). TNF-α expression decreases the presence of amyloid-β plaques in the mouse hippocampus (184), indicating that these cytokines may actually decrease AD pathology. Clearly, more research is needed into the complicated role of inflammatory cytokines in AD patients.

Cholinomimetic drugs, especially AChE inhibitors, have been the first line of defense in AD treatment for many years, and as mentioned above, their benefits are thought to be due to a synaptic increase in ACh or direct stimulation of ACh receptors. However, some of the benefits of these drugs in AD patients may be due to decreases in inflammation in addition to their synaptic actions. Levels of IL-6 are higher in the brains of AD patients—however, this level decreases dose-dependently based on how many months the patients have taken AChE inhibitors (185). Possibly, by acting on microglial α7 nAChRs, cholinergic drugs may decrease the release of inflammatory cytokines and slow neurodegeneration.

Neuroinflammation following neurodegeneration and other neuropathologies (plaques and tangles) likely contributes to clinical impairments in memory (and perhaps other symptoms as well) in both Parkinson’s disease and AD, and speeds progression of these diseases. However, it is also possible that breakdown in the innate immune system, leading to altered glial–neuron responses, contributes to the onset of AD and Parkinson’s in different ways. Dursun et al. (178) compared peripheral cytokine levels in early-onset AD, late-onset AD, mild cognitive impairment, and Parkinson’s disease. Though early-onset AD patients displayed increased levels of inflammatory cytokines, Parkinson’s disease patients exhibited a different profile: IL-1β was increased, but IL-6 was significantly decreased. The interaction between these two cytokines appears to be different in AD and Parkinson’s disease, suggesting that the glial contributions to these diseases may be different.

## Conclusion

Acetylcholine has multiple mechanisms by which it can modulate hippocampal memory: ACh binds directly to neuronal pre- and postsynaptic receptors, initiating downstream neuronal actions, and to receptors on astrocytes and microglia to decrease pro-inflammatory cytokines and increase the release of growth factors like BDNF. ACh can also act *via* the peripheral or central anti-inflammatory pathways by suppressing overactivation of peripheral macrophages or central microglia to indirectly aid memory. Not only does evidence point to multiple effects of ACh on memory processes, there are also multiple effects of ACh on neuroplasticity—specifically, alteration of spine density, synaptic strength, BDNF, and hippocampal neurogenesis. These findings suggest new ways of preventing age-related memory decline and perhaps delaying or preventing the cognitive impairments accompanying neurodegenerative disorders.

## Author Contributions

SVM wrote this manuscript with significant contribution in ideas, structure, and editing from CLW.

## Conflict of Interest Statement

The authors declare that the research was conducted in the absence of any commercial or financial relationships that could be construed as a potential conflict of interest.
